# Saunders’s Pocket Medical Formulary, with an Appendix

**Published:** 1899-04

**Authors:** 


SAUNDERS'S POCKET MEDICAL FORMULARY, with an Appendix. By WILLIAM M. POWELL, M. D., Author of Essentials of Diseases of Children; Associate Editor Annual Universal Medical Sciences, etc. Fifth edition, thoroughly revised. Price, $1.75 net. Philadelphia: W. B. Saunders. 1899.

The fifth edition of this popular little work is made to keep abreast of the times and is consequently thoroughly revised and augmented to bring it in line with the advances that have been made in therapeutics. Many new formulæ have been incorporated, whilst many of the older date and lesser usefulness have been replaced by others of recent origin and established worth. The tables of doses have been modified and amplified in conformity with the change wrought by time and the section on Incompatibles and poisons and antidotes considerably enlarged. Like its predecessors it is worthy of the confidence of the busy practitioner and is to be commended.

